# Determinants of self-efficacy of driving behavior among young adults in the UAE: Impact of gender, culture, and varying environmental conditions in a simulated environment

**DOI:** 10.1016/j.heliyon.2023.e13993

**Published:** 2023-02-24

**Authors:** Praveen Kumar Maghelal, Juan Carlos Flores Lara, Ravindra S. Goonetilleke, Ameersing Luximon

**Affiliations:** a Faculty of Resilience, Rabdan Academy, Abu Dhabi, United Arab Emirates; b Department of Industrial and Systems Engineering, Khalifa University, Abu Dhabi, United Arab Emirates; c Industrial Design, Georgia Tech Shenzhen Institute/Tianjin University (GTSI), Shenzhen, China

**Keywords:** Driving behavior, Virtual reality, Simulation, United Arab Emirates, Road crashes

## Abstract

Research on traffic accidents have acknowledged that human error is the leading cause of road accidents around the world. In the UAE, those aged between 18 and 30 years are involved in the most accidents. As a result, this study examines the perception, attitude and driving behavior of young adults in the UAE. Virtual Reality (VR) was used to examine driving behavior because it offers alternatives to assess driving behavior with a high degree of immersive experience in a safe and replicable environment. Participants drove through a virtual environment that resembled the urban environment of Abu Dhabi in the UAE, which included six traffic events. A sample of 12 females and 27 males also completed a pre and post-simulation questionnaire to report and evaluate their personal driving experience in Abu Dhabi. The volunteer group represented young drivers with limited driving experience and diverse cultural backgrounds. Results indicated that male drivers were less adhering to safe driving behavior compared to females. Even though both males and females exceeded the designated speed limit, males traveled longer distances over the limit. Additionally, it was found that young drivers tend to overestimate their skills with factors like gender, cultural background, and driving experience being key contributors. The results indicate that traffic authorities should take into consideration different approaches in the formulation of policies related to young drivers with periodic reassessment of skills and training to enhance the safety of driving in the UAE and the region.

## 1 Introduction

### 1.1 Background

Sustainable transportation has been emphasized by the United Nations (UN) as a crucial element for success in the implementation of the 2030 Agenda for Sustainable Development, particularly to reach the goals related to health, food security, energy, infrastructure and cities, human settlements, and climate change. The Global Status Report on Road Safety by the World Health Organization [1], reported that every day more than 3500 individuals are killed in road accidents worldwide, adding up to 1.35 million fatalities per year and 50 million additional injured and disabled. Also, road injuries are the leading cause of death for children and young adults below 29 years of age. The 2030 Agenda recognizes this phenomenon as one of the most significant challenges related to sustainable transportation.

Road deaths and injuries in the Middle East have increased in recent years. The region has the third-highest rate of deaths, behind Africa and Southeast Asia [1]. Data from the Minister of Interior (MOI) in the UAE show two people die every day due to road accidents [2]. RoadSafetyUAE [3] reported that young drivers below 30 were responsible for 45% of all road accidents in the country complementing other studies that report that novice drivers between 18 and 24 have double death rates in comparison with older drivers due to road accidents [4]. According to the MOI, the leading causes of accidents are inappropriate driving behavior like speeding, sudden lane changing, lack of following regulation and lack of attention [5]. Also, recent research report that 90% of all accidents are caused by male drivers [2].

Although the number of road crashes have been historically high in the Gulf Cooperation Council (GCC) countries, studies conducted in the region have been scarce, hindering the proper assessment of the magnitude of the problem [6]. Additionally, the UN report targeting road safety emphasis that, “… through better identifying risks and vulnerable areas, gaps in training and training needs could be identified as well. For such training to be effective, customized courses adapted to the stakeholders' needs, policies and practices, could be very useful and is recommended.” [7].

The use of virtual reality allows for affordable driving simulation characterized by a high degree of immersive feeling, allowing the study of this complex phenomenon without exposing participants to risky situations [8,9].

This study examines the driving behavior of young adults using virtual reality, with a particular emphasis on the self-perception of driving behavior. We investigate the change in drivers' responses to varying conditions, change in confidence (measured as efficacy) levels after exposure to the simulation, and the role of indicators of driving behavior on the reported change in their efficacy. Specifically, we examine whether the self-efficacy of the driver’s change pre- and post-driving, and if so, whether it decreased or increased after the simulated driving experience. Following this, we analyze the role of individual and driving behavior and characteristics on the increases or decreases of efficacy of being a safe driver and following traffic rules.

Safe driving relates to a driver’s ability to drive in a road environment and responding to the changing driving environment on the road while following traffic rules. Unsafe drivers generally tend use mobile phones and tailgate while not following rules such as posted speeds and not stopping at pedestrian crossings. Studies report that one determinant of traffic crashes around the world is over-estimating the driver’s perception of being a safe user of the road following traffic rules [10]. Additionally, the United Arab Emirates is one of the GCC countries that report a high percent of expatriates (88%) from multi-cultural backgrounds having varied driving experience, making it an interesting case for this study [[11], [12], [13]]. Additionally, this study makes unique contributions by Ref. [1] investigating the determinants of self-efficacy of young drivers in the UAE, and [2] examining the driving behavior in a multicultural setting of the UAE. The outcome of this study has implications, especially for its multicultural context, as we are living and approaching a multicultural world which will require attention to varying ideologies and their interaction in the society [14]. However, fewer studies have inquired such contextual impact on driving behavior and to our knowledge, none in the middle-eastern region.

Findings of this study will be relevant in the design and implementation of solutions and formulation of policies to support the mitigation of road accidents; and achieve the goals of the 2030 Agenda not only in the UAE but also in other GCC countries and countries with varying cultural demographics, which share geographic, social and cultural similarities with the UAE.

### 1.2 Driving behavior determinants

Driving behavior includes an individual’s response to varying driving conditions. Such behaviors are generally divided into operational, and visual, and physiological-psychological factors [15]. On the contrary, other factors such as individual characteristics, perception and attitudes towards use of the road usually influence driving behavior without the driver’s awareness [16]. Using such factors for deeper analysis of individual differences in driving behavior enables more effective development of road safety policies.

Traditionally, females and males exhibit distinctive behaviors. These differences are linked to biological and psychological factors, and not to experience and differences in capabilities and skills [17]. The relation between gender and risk behavior has been extensively documented. Literature suggests that men are likely to take more risks than women, as a consequence of human evolution [18]. Nevertheless, the willingness of individuals to take risks has been recognized as a core characteristic of personality [19].

A recent study reported that the proportion of young male drivers below 25 who are involved in major road accidents is 1.7 times higher than the rest of the population [20]. Young individuals seeking to satisfy a sense of power, social recognition, and self-esteem tend to take more risks on the roads [21]. Research suggests a lack of awareness in the assessment of the magnitude of risky behavior among teenagers or a contradictory conduct to their knowledge [22]. It is therefore important to understand the level of confidence these young drivers have on their ability to drive safely and follow traffic rules while on the road.

Additionally, environment and culture have significant influence on driving behavior, and some researchers argue that culture plays an important role in safe driving [23]. Also, differences in infrastructure are likely to result in differences in driving behavior. For instance, a study in Qatar found common driving errors irrespective of their ethnicity [24]. Records of car crashes in the emirate of Abu Dhabi between 2012 and 2017 show that Emirati drivers were responsible for 25.5% of all crashes and 39.6% of the fatal crashes in the emirate while Pakistani drivers were responsible for 20.6% of the total accidents and 21.5% of the fatal ones [6].

Based on the well documented fact that most traffic crashes occur due to human actions, Reason et al. (1990) developed the Driving Behavior Questionnaire (DBQ) to measure the concepts of violation, and error, and later included measures of lapses in driver behavior [25]. Several studies have validated these measures and have adopted them to analyze driving behavior in various countries around the world [26,27]. The DBQ consists of 34 items which are divided into four groups (violations, errors, lapses, and other behaviors) The first group contains items about ordinary and aggressive violations in traffic [28]. Ordinary violation is conceptualized as intentional deviation from the legal rules. On the other hand, aggressive violation can be defined as conflictive behavior towards other users. The second group is composed of errors that are decisions that can expose drivers to dangerous situations without breaking any traffic rules. The third group includes a series of lapses which are conceptualized as ill-suited behaviors that usually are related to a lack of concentration on the task The last group consists of items with positive behaviors and other items that generally define other driving behavior.

### 1.3 Distracted driving

Driving demands utmost attention hindering the performance of any secondary task that leads to the redistribution of attention which may have negative consequences while driving [29]. For instance, in the US in 2019, 3142 people were killed, and about 424,000 individuals were estimated to be injured due to distracted driving [30]. Distractions while driving are often classified into visual, manual, and cognitive [31]. Visual distractions are whenever the driver’s sight is off the main task (taking eyes off the road); manual when drivers manipulate devices unrelated to the main task (taking hands off the wheel); and cognitive, whenever attention is withdrawn from driving to assign it to secondary tasks (taking mind off the driving) [32]. Use of mobile phones while driving relates to all three types of distractions and have been extensively investigated.

The use of mobile devices has become a habitual behavior among two- and four-wheel vehicle drivers [2]. Individuals between 18 and 25 tend to use their devices while driving more than any other age group since they have grown up with them [33]. Studies report an increase in accidents by 3 and 4 times when drivers call or text during driving [29]. Use of mobile devices influences the physical and cognitive workloads that lead to a lack of perception and reaction under unexpected events such as pedestrians on road, change on signals or construction on the road [34]. Additionally, studies report that roads with works sites have a higher crash rate during distracted driving [35].

### 1.4 Driving simulation

Simulation offers a controlled and highly replicable setting that can also reproduce complex and risky scenarios [36]. Nevertheless, there are some drawbacks of virtual reality. Most simulators seldom have the capacity to reproduce the vehicle dynamics associated with the road characteristics [37]. Perception of the virtual driving experience can be negatively impacted by simulation sickness, a well-documented but hardly avoidable phenomenon [38]. The effects of simulation sickness include dizziness, drowsiness, headache, nausea, and fatigue and usually appear after long exposure to virtual reality. Age is related to the likeliness to develop this discomfort. While older drivers above 70 are more susceptible [39], young adults are not affected by the spatial cognition issue in the simulator (see Refs. [40,41]).

The use of virtual environments requires significant simplifications in the approach and analyses of each phenomenon [42]. The parameters that are usually used to evaluate driving performance are longitudinal speed control, headway distance control, brake reaction time, lateral steering error, lane departure, etc. [43]. However, it is recommended that, to be valid, the driving simulators have to replicate the environment and the responses in the same way they take place on the road [44].

Validation of simulation occurs in two levels, absolute and relative. Absolute validation is associated with the numerical correspondence between the performance in the virtual environment and the physical world. While, relative validity corresponds to the resemblance between effects of different variations in the simulation [9,45]. Scholars agree that such validity is necessary in order to use a simulator as a research instrument [46]. Comparison between the results of the simulator and the real world can be done in different ways. The most common are self-reported driving behavior, allied health assessments, and on-road tests in vehicles with instrumentation [47]. For over a decade, several studies have validated the similarity in driving performance in the simulator and instrumented vehicles in the real world using driving speed [44].

The use of speed as a measure of driving behavior has increased significantly during the last two decades [37]. Ma et al. [42] assessed driving distraction caused by the devices of 13 mass-produced vehicles in a simulator using distraction indicators such as speed deviation. Meanwhile, Choudhary and Velaga [29] analyzed the effects of texting and phone calls on driving performance of Indian drivers using five scenarios with varying conditions. Their study used speed as an outcome measure to compare driving behavior across these test scenarios. Steinhauser et al. [48] developed a study to assess the influence of emotions on driving behavior by creating visual and auditory stimuli. They used speed variability as a measure to analyze its impact on emotion and attention while driving. The current study, henceforth, used speed as a measure of driving behavior across six test events in a simulated environment.

Specifically, we investigate the driving speed and the self-efficacy of the respondents of being a safe-driver and following the traffic rules. Henceforth, we investigated if: (1) Does driving speed varies by gender and nationality? (2) Do self-perception of being a safe driver and following traffic rules change after driving in the simulated environment? and (3) What are the determinants that relate to the change in their perceptions of being a safe-driver and following traffic rules?

## 2 Method

### 2.1 Participants

For this study, a total of 39 participants from several ethnic backgrounds aged between 19 and 29 years were recruited through snowball sampling. Thirteen were UAE nationals, and the remaining 26 were expatriates of various nationalities. The average age of the participants were 22 years with 12 females and 27 males. While gender differences on driving behavior have been investigated extensively, the differences in national (Emirati) and local have only recently gained interested among researchers [49] The average driving experience of the sample was 3.2 years, representing the novice segment of drivers in the emirate and represents the population at high risk of road crashes as per the recent WHO report [50].

### 2.2 Test protocol

The experimental protocol included three main phases. In the first phase, subjects read and signed a consent form following which, each participant filled a pre-test survey that included a driving behavior questionnaire (DBQ). The second phase involved subject driving in a practice and an actual test virtual environment. The first environment was developed as a training environment where participants were able to test the simulator controls, and were allowed to repeat this session as many times as desired until they felt comfortable with the device. The second simulated environment was the actual test environment wherein each participant drove a distance of 4000 m which included the six test events during the drive. In the last phase of the protocol, participants completed a post-test survey to assess their experience with the driving simulation.

### 2.3 Pre and post questionnaires

The pre-test questionnaire consisted of two sections; the first inquired the socio-demographic information while the following section included question related to their driving behavior. These questions were adapted from the Manchester DBQ [51] to the UAE context and included 34 items rated at a five-point scale (1 = never; 2 = rarely; 3 = occasionally; 4 = often; 5 = almost always). The DBQ items were cataloged into four main categories: violations, errors, lapses, and others as validated and adapted across various countries to analyze driving behavior [[52], [53], [54]]. Violations were defined as an intentional aberration of traffic rules and errors resulting from unexpected behavior related to these rules. On the other hand, lapses are characterized by failures in attention or memory, and others included items that did not fit in the previous three groups. The post-test questionnaire included questions related to the drivers' experience with the simulation during the experiment and about their perception of being a safe driver and follower of traffic rules. Respondents rated themselves from 1 to 5, with 1 being not at all and 5 definitely yes.

### 2.4 Test scenarios

Two virtual environments were developed by scripting in the STISM DRIVE simulator. The first one served as a training environment while the second was the test environment designed to resemble the urban setting of Abu Dhabi with roads having eight lanes, four in each direction, each of 12 feet width. A total of six blocks and five intersections with traffic lights were designed with block size of 650–700 m (average block length in Abu Dhabi), for a total length of 4 km for the test scenario. Traffic flow was adjusted to replicate the peak hour traffic in the city of Abu Dhabi. The signage of designated speed limit of 80 km/h represents the speed on Abu Dhabi arterials while the construction zone was with a reduced speed limit of 40 km/h. Surrounding buildings were primarily mid-rise buildings, with approximately 20 floors and included bus stops on each block with palm trees on the sidewalk as can be observed along the blocks of Abu Dhabi.

Six main events were simulated in the test environment (Table 1). The first was an incoming call that started after 200 m of driving down the road and lasted until 550 m. Participants were able to decide if they wanted to attend to the call. The second event was the traffic light turning amber on the second intersection after 1310 m. The third event was located shortly after the traffic light with a pedestrian crossing the road, a behavior observed often on the streets of Abu Dhabi. The fourth one consisted of a traffic jam with a vehicle swerving into the driver’s lane at 1825 m. The fifth event was an accident on the first lane between 2255 and 2330 m, forcing the driver to merge into another lane. The last event was a section of road under construction, from 2560 to 3020 m. Wherein the fourth lane was unavailable to drive and the speed limit was set to 40 km/h.

**Table 1:** Location of events in the simulation.

**Abbreviation**	**Event**	**Distance [m]**
E1A	1st Phone call begins	200
E1B	1st Phone call ends	550
E2	2nd Traffic light changing to red	1310
E3	3rd Pedestrian crossing the street on a non-allowed area	1370
E4	4th SUV pulls into drivers' lane	1825
E5A	5th Traffic accident on fast lane starts, location of ambulance	2255
E5B	5th Traffic accident on fast lane ends, location of second crashed car	2316
E6A	6th Construction area begins, location of speed sign	2560
E6B	6th Construction area ends	3017

### 2.5 Analysis

Data from the simulator, especially the driving speed was assessed to understand the driving behavior of the participants during the six test events. Average speeds were compared across locals and other nationalities and gender using mean t-tests. Also, survey responses about respondent’s self-perception of being a safe driver and following traffic rules were compared before and after the experiment to assess the self-efficacy across nationalities, gender, driving experience, and individuals with or without other international driving experience (license).

### 2.6 Dependent variable

Change in self-efficacy was assessed through their responses before and after the simulation. Respondents were asked, with a pre- and post-survey, whether they consider themselves to be a ‘safe driver’ and whether they usually ‘follow traffic rules’. Responses ranged from 1 (not at all) to 5 (definitely yes). The average self-efficacy of drivers reduced after the simulation for both safe driver perception and ability to follow traffic (Table 2). While over 40% of the respondents did not report a change in efficacy, 39% and 36% of respondents reported a reduced self-efficacy of being a safe driver and following traffic respectively.

**Table 2:** Measure and change of self-efficacy of drivers.

Self-Efficacy	Simulation^a^		Change in Self Efficacy after Simulation^b^		
	Before	After	*No Change* (*0*∗)	Decrease (1)	Increase (2)
Safe Driver	4.02 (0.99)	3.69 (1.06)	*18 (46%)*	15 (39%)	6 (15%)
Follow Traffic	4.07 (0.98)	3.79 (1.00)	*17 (43%)*	14 (36%)	8 (21%)

a Mean(Std. deviation).

b Cases (percentage of total responses).

∗ Base case.

### 2.7 Independent variables

The individual characteristics of drivers include the age and gender that have shown consistent yet varying relationship with driving perception [55,56]. Other characteristics relate to the region of study. With UAE reporting high expatriate population, we investigated the role of being an expat and a local on change in self-efficacy of driving behavior. Also, the expats in the UAE can obtain a driving license by passing the driving test post training with a registered driving school, or individual holding an active license from selected countries can directly apply for UAE license without any examinations. This lends itself to a situation where the road users in the UAE come with driving experiences from various parts of the world. Therefore, we inquired if each respondent possessed a driving license from a country other than UAE. Finally, longtime users of roads tend to overestimate their driving skills or may become too complacent with their driving behavior [10]. Henceforth, we inquired the tenure of their driving experience and the frequency of them driving in a week.

### 2.8 Driving behavior

The DBQ consisted of 34 items which were divided into four groups (violations, errors, lapses, and other behaviors) (See Ref. [25]). The first group contains items about ordinary and aggressive violations. The responses of these items (nine) were aggregated together as ‘violations’ with Cronbach’s alpha of 0.81, indicating a high internal consistency. The second group is composed of errors. Responses to eleven items were aggregated as the measure of ‘error’ reporting a Cronbach’s alpha of 0.68, indicating moderate internal consistency. The third group includes a series of lapses which consists of eight items reporting a Cronbach’s alpha of 0.69. The last group on positive behaviors and other items aggregated using six items reported a Cronbach’s alpha of 0.52, reporting an acceptable internal consistency [57,58]. While majority of the variables analyzed using for the *t*-test and the regression were measured on Likert-scale, only the combined measured of violation, error, lapses and others were analyzed for normality. Studies have reported that the parametric and the non-parametric assessment of single Likert-scale data do not require a test of normality to conduct the t-tests [59,60]. However, combined measures of such Likert-data can be tested for normality [61].

Table 3 report the skewness ad kurtosis values, which indicate skewness in measures of violation and lapses, and hence were transformed accordingly.

**Table 3:** Normality test for combined Likert-Scale Measures.

				Joint	test
Variable	Obs	Pr(skewness)	Pr(kurtosis)	Adj chi2(2)	Prob > chi2
Violation	39	0.0003	0.0339	13.56	0.0011
Errors	39	0.1091	0.6845	2.93	0.2307
Lapses	39	0.0089	0.0576	8.85	0.012
Others	39	0.4918	0.8654	0.52	0.7726

## 3 Results and discussion

### 3.1 Descriptive analysis

Table 4 reports the means and standard deviation of the demographic, behavior (from both simulation and the DBQ) and the perception of being a safe driver and following traffic rules as reported by the 39 test subjects. The average of the respondents was about 22 years representing the most vulnerable road crashers group. While 33% were local nationals, most of the respondents (69%) were male with majority having attended a driving school in UAE (a requirement of licensing application for new drivers in UAE). While all the test subjects did not report a safe driving behavior in the simulated environment, 64% followed traffic rules in the simulated environment.

**Table 4:** Descriptive analysis of test subjects.

Variable		Obs	Mean	Std. Dev.	Min	Max
Demography						
	Age	39	22.41	2.78	18.0	29.0
	Gender (Male = 2)	39	1.69	0.47	1.0	2.0
	Nationality (1 = UAE)	39	0.33	0.48	0.0	1.0
	Licensed in other country (Yes = 1)	39	0.28	0.46	0.0	1.0
	Attended Driving School in UAE (Yes = 1)	39	0.90	0.31	0.0	1.0
	Driving Frequency	39	3.56	0.94	1.0	4.0
	Driving Experience (in years)	39	0.49	0.51	0.0	1.0
**Perception**						
	Self-Perception of being a safe driver	39	0.69	0.73	0.0	2.0
	Self-Perception of following traffic rules	39	0.77	0.78	0.0	2.0
**Behavior in Simulation**						
	Did not follow traffic rules	39	0.64	0.49	0.0	1.0
	Did not perform safe driving	39	1.00	0.00	1.0	1.0
**Driving Behavior**						
	Violation	39	2.10	0.71	1.2	4.0
	Errors	39	1.71	0.40	1.0	2.8
	Lapses	39	2.03	0.57	1.1	3.9
	Others	39	2.62	0.45	1.5	3.5

**Fig. 1:**
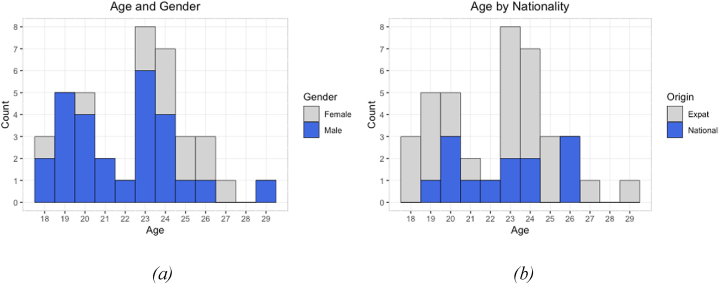
Distribution of age by gender (a) and nationality (b).

Fig. 1a presents the age distribution of participants, varying from 18 to 29 with a global average of 22 years. The classification by gender indicates 12 female subjects with an average age of 24 and 27 males with an average age of 22. On the other hand, the distribution by nationality (Fig. 1b) shows a sample composition of 33% of Emirati subjects with an average age of 22 and 67% of expats with an average age of 22.

**Fig. 2:**
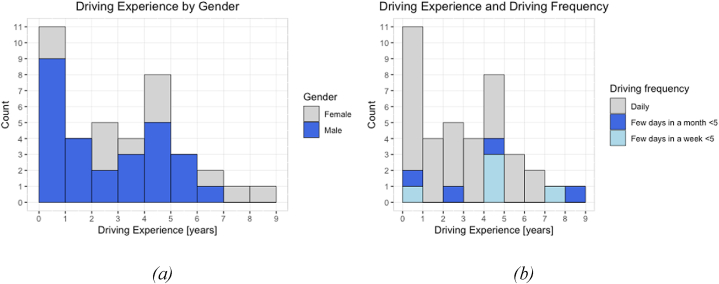
Distribution of driving experience by gender(a) and driving frequency (b).

The sample registered a mean driving experience of 3.5 years. As shown in Fig. 2, in this sample, females have a higher driving experience than males with means of 4.4 and 3 years, respectively; eleven participants reported to have less than one year of experience. The figure additionally shows that 77% of participants reported driving every day.

Only 28% of the subjects reported having a license from another country possibly because most of the respondents are novice drivers. Data shows that 90% of participants reported attending driving school as part of their licensure in the UAE.

### 3.2 Driving behavior

#### 3.2.1 Simulated events

**Fig. 3:**
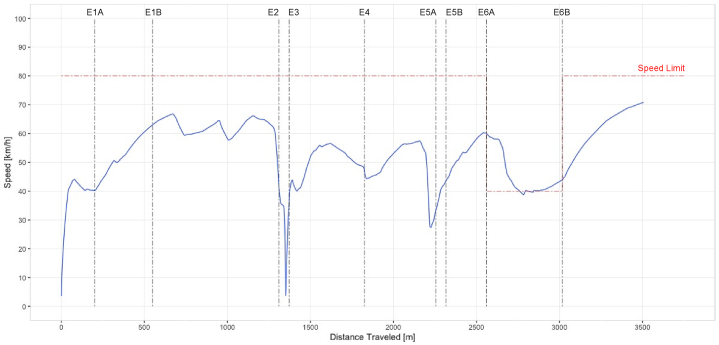
Sample data of Speed vs. distance traveled of a test subject.

The simulator collected driving-related data during the entire distance of the simulated test environment. Variables were measured on distance lapses of 0.5 m for each defined event and its location within the simulation. Driving speed measured in the simulator was assessed for this study. Fig. 3 displays a sample of the speed records of a subject. Vertical lines indicate the distances within the simulated environment where the defined events occurred in the experiment while driving. The red dashed line denotes the speed limit along the road corridor. It is clear that this subject did not exceed the speed limit on the regular zone.

But did so in the construction zone despite the speed decrement between E6A and E6B to 40 km/h. Another two relevant decrements can be noticed between E2 and E3 and before E5A. The first one indicates how the subject did not stop at the traffic light (E2) but suddenly braked to avoid colliding with the pedestrian on E3. The second speed reduction indicates awareness of the road accident. Finally, the sight speed decrement on E4 shows that the driver reacted to the vehicle pulling into their driving lane.

### 3.3 Response to varying conditions

Average speed was calculated for the three events (E2 to E4) at 5 m before and after occurrence of the event, while for the other three events (E1, E5, and E6) the average speed was calculated from the start to the end of the event. Table 5 summarizes the results of mean speed for the global sample and groups classified by gender and nationality.

**Table 5:** Mean values of speed for each event classified by groups.

Sample	Mean Speed [km/h]						Speeding Instances
	E1	E2	E3	E4	E5	E6	
Global	61.78	50.02	53.03	58.62	54.75	51.46	2.03^b^
Males	63.14^a^	50.88	53.62	60.50	55.13	51.79	2.41
Females	58.73^a^	48.08	51.72	54.38	53.89	50.72	1.17
Nationals	64.60	50.83	57.20	61.55	63.27^b^	53.73	2.04
Expats	60.37	49.61	50.95	57.15	50.49	50.33	2.00

a p<0.10;

b p < 0.05.

Drivers' speed for all six events were analyzed across different gender and nationality. A series of two-sample t-tests for means (of speed) for the six events reported that the nationals reported a higher average speed for all the events. The number of instances of speeding in non-construction zone was compared across gender and nationalities. On average the number of instances that males over speeded in the simulated environment was higher than females (p < 0.05). However, only the means of E1 (call) and E5 (accident) are statistically different. The p-value of 0.089 for E1 indicates that the mean speed was marginally different. It was observed that the average speed of expats was lower than the locals by about 13 kmph for E5 (accident) indicating higher level of awareness by the expats during the accident.

The gender difference of driving speed across the six events report no statistical significance except for the use of mobile (E1). Females reported a lower mean which was statistically significant at p < 0.10. Although marginal, this difference of male driving at higher average speed aligns with other studies that men are more inclined to take risks during driving, justified as a consequence of human evolution (Buss, 2005).

### 3.4 Self-efficacy of driving

This study analyzes if and how the confidence level of drivers changed before and after the simulation by comparing the results of the two questions included in both pre and post-simulation questionnaires. The sample was classified in groups according to gender, nationality, driving experience, and the possession of more than one license. The mean driving experience (3.5 years) was used to divide the sample into groups above and below the mean. T-tests were performed to analyze the means of the subjects' self-perception of being a safe driver before and after the test.

The global sample, which includes responses from all the subjects, experienced a decrement of safe driving self-perception (Table 6). Before the simulation, most drivers scored themselves with a mean of 4.02, and after the simulation, the mean reduced to 3.69. This result supports the claim of several studies that drivers tend to overestimate their driving abilities. Females reported a significant decrement in the mean values among all the groups. This indicates the re-assessment of females of their confidence of being a safe driver leading to overall mean by 0.75. Additionally, those holding license from other countries as well reported highest average of their efficacy to be a safe driver. This reduced significantly after the simulation with p < 0.05. This indicates that possibly their driving experience (represented by license from other country) is different from driving in the UAE and hence a simulation representative of Abu Dhabi with various elements of distraction led to reduction in their efficacy of being a safe driver.

**Table 6:** Results of paired *t*-test of self-perception of being a safe driver before and after the simulation.

Group	Safe Driving Before Simulation	Safe Driving After Simulation	Difference	t-value	p-value
Global Sample	4.02	3.69	0.33	1.83	0.073
Nationals	4.07	3.61	0.46	1.19	0.255
Expats	4.00	3.73	0.27	1.37	0.183
**Females**	**4.00**	**3.25**	**0.75**	**2.13**	**0.055**
Males	4.03	3.88	0.15	0.72	0.476
Driving experience >mean	4.16	3.79	0.37	1.38	0.185
Driving experience <mean	3.90	3.60	0.3	1.18	0.249
**Holds a license from other country**	**4.36**	**3.73**	**0.63**	**2.28**	**0.045**
Does not hold a license from other country	3.89	3.68	0.21	0.95	0.352

The difference in self-perception of following the traffic rules among the expats, females and those holding license from other country reports a reduction in the self-efficacy after the simulation (Table 7). While other groups reported reduced means, they were not statistically significant. As with the perception of being a safe driver, females reported a reduction of 0.75 in their ability to follow traffic rules after the simulation. This indicates the various events in the test environment made them re-assess their skills and ability to follow traffic rules. Expats reported the lowest mean prior to the simulation indicating a lack in self-confidence to follow the traffic rules of UAE. Prior research report difficulty in understand rules and sign boards (sometimes in Arabic) that has impacted the ability to follow traffic rules. Of the 39 respondents 11 expats reported holding license from other country, while the other 28 expats and national did not hold any other license. This change is confidence can be related to the experience of the expats with the driving in the UAE.

**Table 7:** Results of paired *t*-test of self-perception of following traffic rules before and after the simulation.

Group	Following Rules Before Simulation	Following Rules After Simulation	Difference	t-value	p-value
Global Sample	4.08	3.79	0.29	1.64	0.109
Nationals	4.38	4.15	0.23	0.58	0.569
**Expats**	**3.92**	**3.61**	**0.31**	**1.77**	**0.088**
**Females**	**4.33**	**3.58**	**0.75**	**2.01**	**0.068**
Males	3.96	3.88	0.08	0.41	0.678
Driving experience >mean	4.05	3.78	0.27	1.15	0.262
Driving experience <mean	4.10	3.80	0.3	1.14	0.267
**Holds a license from other country**	**4.09**	**3.54**	**0.55**	**2.20**	**0.051**
Does not hold a license from other country	4.07	3.89	0.18	0.81	0.421

### 3.5 Determinants of change of self-efficacy

The change in self-efficacy of the young drivers was examined using determinants such as individual and driving behavior characteristics. Latent measures derived from the DBQ were grouped as driving behavior characteristics while the individual’s gender, nationality. Driving experience, and licensing in other countries were grouped as individual characteristics. Results from the Multinomial logistic regression indicate male, licensed in another country, taking driving lessons in the UAE, higher driving frequency, violations-related driving behavior, and self-reporting by respondents to not follow rules are significant determinants of change in efficacy of perception of being a safe driver and following traffic rules (Table 8).

**Table 8:** Results of change in self-perception before and after the simulation using Multinomial Logistic Regression.

	Perception of Safe Driver		Perception of Following Traffic Rules	
	Decrease in Efficacy	Increase in Efficacy	Decrease in Efficacy	Increase in Efficacy
Gender (Male 1)	0.170 (1.075)	22.174 (5.503)***	−4.357 (1.582)***	−2.857 (1.399)**
Licensed in other country (Yes = 1)	1.096 (1.056)	−33.242 (4.032)***	0.408 (1.037)	−2.124 (3.098)
Attended Driving School in UAE (Yes = 1)	19.826 (1.027)***	−16.931 (2.426)***	0.444 (1.783)	13.852 (1.057)***
Driving Frequency	0.370 (0.450)	−1.665 (1.527)	−0.791 (0.453)*	0.545 (0.773)
National (Yes = 1)	0.658 (1.114)	0.679 (1.570)	−2.523 (1.047)**	−1.159 (1.259)
Violation	−14.111 (5.704)**	−19.168 (8.064)**	12.786 (7.373)*	−10.356 (8.714)
Lapses	7.0112 (6.195)	4.090 (12.723)	−0.155 (6.760)	0.879 (6.146)
Errors	0.625 (1.594)	0.206 (2.406)	0.401 (1.382)	−1.401 (2.630)
Not Follow Rules	–	–	3.705 (1.460)**	3.593 (2.395)
Obs	39		39	
df	15		20	
AIC	78.904		93.381	
BIC	103.857		126.652	
LR Chi2(16)	30.06		431.93	
Prob > chi2	0.0177		0.000	
Psuedo R2	0.381		0.3511	

Base Case: No change in Efficacy;

∗∗∗ p < 0.01;

∗∗ p < 0.05;

∗ p <0.10.

The perception of being a safe driver reduced for individuals who took driving lessons to meet the requirement to qualify for the UAE driving license. Violation-related behavior reported a negative relation with decrease in their self-efficacy of being a safe driver. While males reported a positive relation with increase in self-perception of being a safe driver, being licensed in other country, taking driving lessons in the UAE, and those reporting a driving behavior that is in violation of safe driving behavior reported a negative relation with increase in the perception in comparison to those reporting no change in perception before and after the simulation.

The perception of following traffic rules while driving reported a negative relation with male drivers, those with higher driving frequency, and being a national. Driving behavior that is in violation and individuals not following rules in the simulation reported a positive relation with decrease in efficacy of following traffic rules in comparison to those reporting no change in efficacy. Increase in efficacy of following traffic rules compared to no change in efficacy reported a negative relation with being a male and a positive relation with those having attended driving school in the UAE.

While male drivers report an increase in efficacy of being a safe driver in comparison to those with no change, they report a negative relation with increase or decrease in efficacy of following traffic rules in comparison to those reporting no change. Studies report that men pose higher risk of accidents than women [62]. However, there is little explanation for the same. This study helps to entangle this by demonstrating that men tend to over-estimate their efficacy of being a safe driver. This is supported by two outcomes of this study. Firstly, the mean perception of being a safe driver among men do not report a significant change before and after the simulation. While this indicates no impact of the stimuli (simulation) on their perception, it also indicates that their self-perception of being a safe driver was high and did not change. Secondly, this could also explain the negative relation of decrease in efficacy of following traffic rules because women tend follow traffic rules better than men [63]. However, the negative relation of increase in efficacy in men in comparison to those reporting no change is contrary to assumption and may require more investigation.

Nationals and expats from countries whose licenses are not eligible for direct conversion have to attend minimum required classes with a registered driving school for licensing in the UAE. Those receiving driving lessons report a negative relation with increase in efficacy and positive relation with decrease in efficacy of being a safe driver in comparison to those reporting no change in efficacy. This indicates that receiving driving lesson in comparison to those who don’t helps drivers to not over-estimate their perception of being a safe driver. While this requires empirical proof, the results suggest that taking driving lesson and attending driving schools helps individuals not over-estimate their driving skills. This is substantiated by the positive relation with increase in efficacy to follow traffic rules.

Violation-related driving behavior reported an expected relation with increase in efficacy of safe driving (negative) and decrease in efficacy of following traffic rules(positive) indicating that those who engage in violation-related driving behavior do not report increase in perception of being a safe driver while reporting a positive relation with decrease in following traffic rules. However, the negative relation of violation-related behavior with decrease in efficacy of being a safe driver in comparison those that report no-change can be speculated as an outcome of self-realization of their own risky behavior as user of the road. However, more research to substantiate this proposition is needed.

## 4 Conclusion and implications

This study examined the driving behavior of young adults in the United Arab Emirates (UAE) and their change in self-efficacy of being a safe driver and following traffic rules. The analysis of how drivers' responses change with varying conditions showed that males and nationals registered higher mean speed values in the six events. In contrast, females reached values below the mean. In addition, trends show that males drove at a higher speed on average during the whole driving test; this is consistent with the results of other experiments that highlight the likeliness of males to perform riskier driving behavior [62].

The analysis of change in self-efficacy report females and expats were more affected by their performance in the driving test. These groups, along with the holders of more than one license, registered the greatest decrements in the mean values of self-perception of being a safe driver and following the traffic rules. The analysis of self-perception vs. behavior exposed relevant contrasts between the participant’s responses in the questionnaires and the measured performance in the driving test. Females and expats rated themselves with lower scores than males and nationals after the driving test. This suggests that they considered they did not follow the traffic rules enough nor were safe drivers.

Nevertheless, the driving behavior in the simulator revealed that 77% of males exceed the speed limit against 33% of females and 69% of nationals compared to 61% of expats. The analysis also exhibited that 100% of participants with higher experience above the mean exceeded the limit. In relation to the assessment of being a safe driver, it was determined that 100% of participants exceeded the speed limit on the road works section. In summary, the results from the pre-post simulation clearly indicate that young drivers, particularly males, tend to overestimate their driving skills since their self-evaluation resulted in values that evidenced their consideration of having performed better than what they actually did.

The perception analysis indicates that males tend to over-estimate their perception of safe user of the road. Hence special attention should be given to reduce reckless driving, especially with speeding and use of mobile devices. The Department of Licensing and Registration, can accumulate such violation data to require individuals to take more driving lessons before renewal of their license. In fact, periodical testing of driving behavior, although not a driving test, can be introduced to reduce the possibility of unsafe driving behavior and adherence to traffic rules and regulation. More importantly, it can reduce the over-estimation of the perception of their own skills and ability to be safe users of the road. A similar virtual setup can be developed that use advanced immersive simulated environment to test and assess the skills of the driver regularly.

The study results provide evidence that there is a need for constant assessment of young drivers' skills. It has been demonstrated that young drivers overestimate their skills, and factors like gender, cultural background, and driving experience contribute to this. Results and evidence from other research indicate that driving education should have a gender distinction [22]. Statistics from the UAE show that male drivers cause most accidents, and this research confirms the behavior differences between males and females. Transportation professionals and decision-makers use the outcome of similar studies in the region to propose gender and nationality based approaches for new policies. For instance, Abu Dhabi in the UAE could be the initial testbed to implement policies related to re-assessment of driving skills among young adults and align these assessments to be gender and ethnicity specific without running the risk of such actions leading to discriminatory ideas. Also, specific needs of the expats to help them understand traffic rules and signs better can improve the overall safety of the road and its users.

This study has its limitations that requires further research. Firstly, the study did not analyze comparative behavior of driver’s behavior in simulated and the real world environment. While a driving simulation is a validated tool, a more immersive environment and its comparison to real world environment can provide higher validity to such studies [64]. Also, the number of test events could be introduced across different driving test environments, i.e., two or more simulated environments, or a test environment (control-group) with more realistic scenario can be used for effective comparison. As for this study, following on other simulated driving environment studies that report eye-strain, nausea, discomfort and disorientation, the length of driving environment was restricted to 4 kms in total [65,66].

Secondly, this study was conducted during the COVID lockdown in Abu Dhabi. Hence the participants were restricted to university students with a mean age of 22 years. While this helps this study to focus on the most vulnerable age group, a more representative population can provide better insights about driving behavior and the change in self-efficacy. Finally, while the DBQ inquired about various driving behavior, its validation with actual driving records can help relate the self-reported behavior to observed behavior in simulation to actual behavior in real-world. This could be data intensive, yet useful in developing policies that specific to the region and its population.

Barring these limitations, this study provides important implications for policymakers of Abu Dhabi and other countries in the region.

1. Males overestimated their abilities in the simulated environment compared to female participants (aged between 19 and 29) and male young adults, the most vulnerable group, should be re-assessed for their driving skills.
2. Perception of being a safe drive and to follow traffic rules reduced when the subjects drove through a simulated environment rather than just inquired prior to actual or simulated driving. Since self-perception responses have an inherit disadvantage, using them as tools for policy making have been debated. Especially with the availability of virtual reality, the use of it for transportation safety research with more advanced immersive simulation approach should be encouraged.
3. Re-assessment of self-efficacy during license renewals can be implemented to improve and assert the confidence levels of drivers and help improve the safety of all the road users.

## Author contribution statement

Praveen Maghelal: Conceived and designed the experiments; Analyzed and interpreted the data; Contributed reagents, materials, analysis tools or data; Wrote the paper.

Juan Carlos Flores Lara: Conceived and designed the experiments; Performed the experiments; Analyzed and interpreted the data; Contributed reagents, materials, analysis tools or data; Wrote the paper.

Ravindra Goonetilleke: Conceived and designed the experiments; Analyzed and interpreted the data; Wrote the paper.

Ameersing Luximon: Analyzed and interpreted the data.

## Funding statement

This research did not receive any specific grant from funding agencies in the public, commercial, or not-for-profit sectors.

## Data availability statement

The data that has been used is confidential.

## Declaration of interest’s statement

The authors declare no conflict of interest.
