# Supplementary material for: Determinants of self-efficacy of driving behavior among young adults in the UAE: Impact of gender, culture, and varying environmental conditions in a simulated environment
Source: Heliyon. 2023 Feb 24;9(3):e13993. doi: 10.1016/j.heliyon.2023.e13993 (PMC10006465; doi:10.1016/j.heliyon.2023.e13993)
Supplement: Multimedia component 1 [file mmc1.pdf]

DRIVING BEHAVIOR QUESTIONNAIRE (DBQ) AND PERCEPTION OF AUTONOMOUS VEHICLES (AVS)

Date: ______________ Subject Number: ______________

The information you provide is anonymous and will be used solely for this study.

Section 1 Demographic Data

1.1. Age

Below or equal to 20 • 21-30 • 31-40 • 41-50 • 51-60 • Over 60 •

1.2. Gender Female • Male •

1.3. Nationality ___________

1.4. What is your designation?

Student • Faculty • Staff • Other: ______________

1.5. Do you have a driving license? Yes • No •

1.6. For how long have you had a license? ___________

1.7. Do you have driving permit in more than once country? Yes • No •

1.8. Did you attend driving school? Yes • No •

1.9. How often do you drive?

Few days in a month (<5) • Once a week • Few days in a week (<5) • Daily •

1.10. Have you received any traffic-related fine in the last year? Yes • No •

1.11. How many? ___________

1.12. Have you been involved in a traffic accident in the last year? Yes • No •

1.13. How many? ___________

Section 2 Driving Behavior

Based on your personal experience while driving during the last year, please indicate how frequently you performed each of the following items according to the following six-point scale:

0 = never, 1 = hardly ever, 2 = occasionally, 3 = quite often, 4 = frequently, and 5 = nearly all the time.

ITEM	0	1	2	3	4	5
2.1. Maintain a large distance between myself and the driver in front of me	•	•	•	•	•	•
2.2. Slow down when approaching intersections even when the light is green	•	•	•	•	•	•
2.3. Press the wrong pedal	•	•	•	•	•	•
2.4. Forget to use my turning signal	•	•	•	•	•	•
2.5. Drive especially close to the car in front as a signal to its driver to go faster or get out of the way	•	•	•	•	•	•
2.6. Cross a junction knowing that the traffic lights have already turned red	•	•	•	•	•	•
2.7. Ignore the speed limits late at night or early in the morning	•	•	•	•	•	•
2.8. Ignore the speed limits on a highway	•	•	•	•	•	•
2.9. Have an aversion to a particular class of road user and indicate your hostility by whatever means you can	•	•	•	•	•	•
2.10. Become impatient with a slow driver in the outer lane and overtake on the inside (right) lane	•	•	•	•	•	•

0 = never, 1 = hardly ever, 2 = occasionally, 3 = quite often, 4 = frequently, and 5 = nearly all the time

ITEM	0	1	2	3	4	5
2.11. Get involved with unofficial "races" with other drivers	•	•	•	•	•	•
2.12. Angered by another driver's behavior, you give chase with the intention of giving him/her a piece of your mind	•	•	•	•	•	•
2.13. Sound your horn to indicate your annoyance to another driver	•	•	•	•	•	•
2.14. Stay in a lane that you know will be closed ahead until the last minute before forcing you into the other lane	•	•	•	•	•	•
2.15. Attempt to overtake someone that you not noticed to be signaling a left/right turn	•	•	•	•	•	•
2.16. Miss "give way" signs and narrowly avoid colliding with traffic having right of way	•	•	•	•	•	•
2.17. Fail to notice that pedestrians are crossing when turning into a side street from a main road	•	•	•	•	•	•
2.18. Queuing to turn right/left onto a main road, you pay such close attention to the mainstream of traffic that you nearly hit the car in front	•	•	•	•	•	•
2.19. On turning right/left nearly hit a two-wheeler who has come up on your inside	•	•	•	•	•	•
2.20. Fail to check your rear-view mirror before pulling out or changing lanes, etc.	•	•	•	•	•	•
2.21. Underestimate the speed of an oncoming vehicle when overtaking	•	•	•	•	•	•
2.22. Apply sudden brakes on a slippery road, or steer wrong way in a skid	•	•	•	•	•	•
2.23. Get into the wrong lane when approaching a roundabout or a junction	•	•	•	•	•	•

0 = never, 1 = hardly ever, 2 = occasionally, 3 = quite often, 4 = frequently, and 5 = nearly all the time

ITEM	0	1	2	3	4	5
2.24. Misread the signs and exit from the roundabout on the wrong road	•	•	•	•	•	•
2.25. Forget where you left your car in the park	•	•	•	•	•	•
2.26. Hit something when reversing that you had not previously seen	•	•	•	•	•	•
2.27. Attempt to drive away from the traffic lights	•	•	•	•	•	•
2.28. Switch on one thing such as headlights, when you meant to switch on something else, such as wipers	•	•	•	•	•	•
2.29. Intending to drive to destination A and, you “wake up” to find yourself in destination B, because the latter is your more usual destination	•	•	•	•	•	•
2.30. Realize you have no clear recollection of the road along which you have been traveling.	•	•	•	•	•	•
2.31. Have difficulty adjusting the controls (e.g., temperature, radio) because it’s too distracting	•	•	•	•	•	•
2.32. Talk or text someone on my cellphone	•	•	•	•	•	•
2.33. Find if too distracting to talk with the passengers	•	•	•	•	•	•
2.34. When driving with friends the way you drive changes	•	•	•	•	•	•

Restricted

Section 3 Perception of Autonomous Vehicles

Based on your knowledge and perception about autonomous vehicles (AVs), please indicate what is requested on the following items.

3.A *State your agreement or disagreement with the following statements regarding AVs, according to the following scale:*

1 = strongly disagree, 2 = somewhat disagree, 3= neither agree nor disagree, 4 somewhat agree, and 5 strongly agree

ITEM	1	2	3	4	5
3.1. They would reduce the number of car accidents	•	•	•	•	•
3.2. They would be safer than non-autonomous vehicles	•	•	•	•	•
3.3. They would reduce my travel time	•	•	•	•	•
3.4. They will allow me to spend time on other activities	•	•	•	•	•
3.5. They would be environmentally friendlier	•	•	•	•	•
3.6. They would consume less fuel	•	•	•	•	•

3.B *If you used an AV, how concerned or unconcerned would you be about the following issues?*

1 = unconcerned, 2 = neutral, 3 = concerned, 4 = very concerned

ITEM	1	2	3	4
3.7. Being legally and financially responsible of the car involved in an accident or makes mistakes	•	•	•	•
3.8. The ability of your car to perform safely in all conditions	•	•	•	•
3.9. Vehicle security (e.g. hackers taking control of the car)	•	•	•	•
3.10. Data privacy (e.g. being able to have your car's location and destination tracked)	•	•	•	•

3.C *I would want to use an AV under the following conditions*

1 = strongly disagree, 2 = somewhat disagree, 3= neither agree nor disagree, 4 somewhat agree, and 5 strongly agree

ITEM	1	2	3	4	5
3.11. When I am tired or fatigued	•	•	•	•	•
3.12. When driving is boring and monotonous	•	•	•	•	•
3.13. When traffic is congested	•	•	•	•	•
3.14. In situations in which I feel uncomfortable driving	•	•	•	•	•

3.D *If I used an AV, I would be comfortable and/or uncomfortable allowing it to…*

1 = very uncomfortable 2 = somewhat uncomfortable, 3 = neither uncomfortable nor comfortable, 4 = somewhat comfortable, and 5 very comfortable

ITEM	1	2	3	4	5
3.15. Stay within the lane by itself	•	•	•	•	•
3.16. Navigate itself to a desired location	•	•	•	•	•
3.17. Comply with the traffic laws by itself	•	•	•	•	•
3.18. Control speed by itself	•	•	•	•	•
3.19. Avoid collisions with other vehicles and road users by itself	•	•	•	•	•
3.20. Change lanes by itself	•	•	•	•	•
3.21. Follow the vehicle ahead at a safe distance by itself	•	•	•	•	•

3.E *How likely would you be able to engage the following activities when driving an AV*

1 = very unlikely, 2 = unlikely, 3 = neither likely nor unlikely, 4 = somewhat likely, and 5 = very likely

ITEM	1	2	3	4	5
3.22. Observing the scenery	•	•	•	•	•
3.23. Interacting with other passengers	•	•	•	•	•
3.24. Eating/drinking	•	•	•	•	•

1 = very unlikely, 2 = unlikely, 3 = neither likely nor unlikely, 4 = somewhat likely, and 5 = very likely

ITEM	1	2	3	4	5
3.25. Using personal devices	•	•	•	•	•
3.26. Resting	•	•	•	•	•
3.27. Reading	•	•	•	•	•
3.28. Grooming	•	•	•	•	•
3.29. Sleeping	•	•	•	•	•

3.F *Willingness to pay for AV technology: Would you be more, less or the same for…*

1 = less, 2 = same, 3 = more, and 4 = a lot more

ITEM	1	2	3	4
3.30. A partially automated car compared to the same car without automation?	•	•	•	•
3.31. Road infrastructure that would help to an AV function more effectively	•	•	•	•
3.32. Special training and licensing procedures to put in place to use AVs?	•	•	•	•
