# Supplementary material for: Determinants of self-efficacy of driving behavior among young adults in the UAE: Impact of gender, culture, and varying environmental conditions in a simulated environment
Source: Heliyon. 2023 Feb 24;9(3):e13993. doi: 10.1016/j.heliyon.2023.e13993 (PMC10006465; doi:10.1016/j.heliyon.2023.e13993)
Supplement: Multimedia component 2 [file mmc2.pdf]

POST-TEST DEBRIEFING QUESTIONS

Date: ______________ Subject Number: ______________

The information you provide is anonymous and will be used solely for this study.

1. How do you feel after your experience with the simulator?

2. What part of your experience made you feel like you were driving?

3. What part of your experience detracted you from feeling like you were driving down the street?

4. Do you consider that this experience was more or less stressful than your daily driving?

5. If you were going to change something about the experience you just had in the simulator, what would that be?

6. Please rank the following perception in how closely your simulation experience represented your experience as a driver in the UAE

Item	Very much	Somewhat	Not very much	Not at all
6.1 Surrounding Activity Level	•	•	•	•
6.2 Sequential Experience	•	•	•	•
6.3 Speed	•	•	•	•
6.4 Visuals	•	•	•	•
6.5 Roads	•	•	•	•
6.6 Overall Street Environment	•	•	•	•
6.7 Buildings	•	•	•	•
6.8 Trees	•	•	•	•

7. How would you rate your perception of the following for conventional (self-driving) and for autonomous driving?

Driving Behavior	Self-Driving					Autonomous-Driving				
	1	2	3	4	5	1	2	3	4	5
7.1 Perception of Safety (feeling)	☐	☐	☐	☐	☐	☐	☐	☐	☐	☐
7.2 Comfort of driving	☐	☐	☐	☐	☐	☐	☐	☐	☐	☐
7.3 Your trust on vehicle's response to the environment	☐	☐	☐	☐	☐	☐	☐	☐	☐	☐
7.4 Control of vehicle speed	☐	☐	☐	☐	☐	☐	☐	☐	☐	☐
7.5 Control of breaking	☐	☐	☐	☐	☐	☐	☐	☐	☐	☐
7.6 Overtaking other vehicles	☐	☐	☐	☐	☐	☐	☐	☐	☐	☐
7.7 Concentration on the road	☐	☐	☐	☐	☐	☐	☐	☐	☐	☐
7.8 Notice signals and signs	☐	☐	☐	☐	☐	☐	☐	☐	☐	☐
7.9 Your observation of the built environment (buildings, trees, streetlamps)	☐	☐	☐	☐	☐	☐	☐	☐	☐	☐
7.10 Confidence with use of cell phone during driving	☐	☐	☐	☐	☐	☐	☐	☐	☐	☐
7.11 Your confidence of maneuvering the environment	☐	☐	☐	☐	☐	☐	☐	☐	☐	☐

1- Highly Negative; 2- Negative; 3 - Neutral; 4- Positive; 5 - Highly positive
